# An Adaptation Multi-Group Quasi-Affine Transformation Evolutionary Algorithm for Global Optimization and Its Application in Node Localization in Wireless Sensor Networks

**DOI:** 10.3390/s19194112

**Published:** 2019-09-23

**Authors:** Nengxian Liu, Jeng-Shyang Pan, Jin Wang, Trong-The Nguyen

**Affiliations:** 1 College of Mathematics and Computer Science, Fuzhou University, Fuzhou 350116, China; lylnx@fzu.edu.cn; 2 Fujian Provincial Key Lab of Big Data Mining and Applications, Fujian University of Technology, Fuzhou 350118, China; jinwang@csust.edu.cn (J.W.); vnthe@hpu.edu.vn (T.-T.N.); 3 College of Computer Science and Engineering, Shandong University of Science and Technology, Qingdao 266590, China; 4 Hunan Provincial Key Laboratory of Intelligent Processing of Big Data on Transportation, School of Computer & Communication Engineering, Changsha University of Science & Technology, Changsha 410000, China; 5 Department of Information Technology, University of Manage and Technology, Haiphong 180000, Vietnam

**Keywords:** differential evolution, multi-group, quasi-affine transformation evolutionary algorithm, global optimization, distance vector-hop, node localization, wireless sensor networks

## Abstract

Developing metaheuristic algorithms has been paid more recent attention from researchers and scholars to address the optimization problems in many fields of studies. This paper proposes a novel adaptation of the multi-group quasi-affine transformation evolutionary algorithm for global optimization. Enhanced population diversity for adaptation multi-group quasi-affine transformation evolutionary algorithm is implemented by randomly dividing its population into three groups. Each group adopts a mutation strategy differently for improving the efficiency of the algorithm. The scale factor F of mutations is updated adaptively during the search process with the different policies along with proper parameter to make a better trade-off between exploration and exploitation capability. In the experimental section, the CEC2013 test suite and the node localization in wireless sensor networks were used to verify the performance of the proposed algorithm. The experimental results are compared results with three quasi-affine transformation evolutionary algorithm variants, two different evolution variants, and two particle swarm optimization variants show that the proposed adaptation multi-group quasi-affine transformation evolutionary algorithm outperforms the competition algorithms. Moreover, analyzed results of the applied adaptation multi-group quasi-affine transformation evolutionary for node localization in wireless sensor networks showed that the proposed method produces higher localization accuracy than the other competing algorithms.

## 1. Introduction

Over the last few decades, global optimization problems have attracted a lot of research interest [1]. Many optimization algorithms have been developed based on inspiration from natural phenomenon, e.g., biological, swarm, physical aspects that are known as natural-inspired intelligent algorithms [2]. The natural-inspired smart algorithms have been widely applied to solve optimization problems successfully [3,4,5]. Genetic algorithm (GA) [6], particle swarm optimization (PSO) [7], differential evolution (DE) [8], ant colony optimization (ACO) [9], Hierarchical archive based mutation strategy with depth information of evolution (HARD-DE) [10] artificial bee colony optimization (ABC) [11], firefly algorithm (FA) [12], bat algorithm (BA) [13], fireworks algorithm (FWA) [14] and quasi-affine transformation evolution algorithm (QUATRE) [15], and cat swarm optimization (CSO) [16] are examples of such algorithms.

The natural-inspired algorithms have been proving robust in delivering optimal global solutions and assisting in resolving the limitations encountered in traditional methods [17]. The optimization process of the natural-inspired intelligent algorithms usually begins with generating a set of randomly initialized agents that combined, immigrated, or evolved over a predefined number of generations. Theses algorithms can be an efficient way to produce acceptable solutions by trial and error to a complex problem in a reasonably reasonable time [18]. There are two algorithms of the algorithms as mentioned above, PSO and DE have been paid much attention and widely used in diverse fields of science and engineering because of their simple and powerful. 

The QUATRE [15] algorithm elaborated its relationship with PSO and DE algorithms. The QUATRE considered as the parameter-reduced or enhanced DE algorithm that conquers representational or positional bias of the DE algorithm. Several variants of the QUATRE algorithm were represented by the conventional notation “QUATRE/x/y” that is similar to the notation of DE “DE/x/y/z” [15,19]. 

The QUATRE algorithm is considered as a robust algorithm [5,20,21,22] with the advantages, e.g., simplicity, a few setting control parameters, easy implementation, and outputting excellent performance. However, similar to other stochastic optimization algorithms, QUATRE algorithm also exists some drawbacks when dealing with complicated problems such as the premature convergence and search stagnation. Some other QUATRE variants have been proposed to alleviate these weaknesses and to enhance the QUATRE’s performance. C-QUATRE aimed to improve the QUATRE’s performance by partitioning the entire population randomly into two groups that evolve the individual who loses in pairwise competition between two groups [20]. For S-QUATRE [21], it partitions the entire population into two groups, i.e., the better group and the worse group using sort strategy, and its individuals only changed in the worse group. Both of these variants used bi-group approaches and employed one mutation strategy to enhance the QUATRE’s performance. Several algorithms introduced the enhanced population diversity based on dividing the entire population into some groups or subpopulations to improve their performance, such as MPADE [23], PPSO [24], and PCSO [25]. In QUATRE variants, the mutation schemes and control parameters (e.g., scale factor) play essential roles in the optimization performance of solving problems due to it would offer unusual exploration and exploitation abilities that caused to methods performance [15]. The searching ability and convergence speed are affected by a scaling factor. The experimental results [19,20] also recommended that setting the scaling factor to a specific constant number could produce functional optimization problems. However, the solution to different practical engineering problems, manual tuning of the control parameters with the appropriate value would be complicated and difficult implementing configuration. To avoid manual tuning of the control parameters, different categorizations of parameter adaptation techniques have been presented for other evolutionary algorithms in [26,27].

In this paper, motivated by multi-population approach and parameter adaptation technique, we propose a novel adaptation multi-group QUATRE algorithm (AMG-QUATRE) with an adaptation scale factor and adopting each group with different mutation scheme to overcome the mentioned weakness of the QUATRE. We evaluate the proposed algorithm by testing CEC2013 benchmark set, and a practical problem of node localization in wireless sensor networks (WSN). The simulation results show that the proposed AMG-QUATRE outperforms the competition algorithms, and improves localization accuracy of the distance vector-hop (DV-Hop) algorithm. Contributions behinds in this paper are as follows.

- A proposed novel AMG-QUATRE algorithm overcomes the deficiencies of the original QUATRE.
- Full use of different mutation strategies along with proper parameters makes a better trade-off between exploration and exploitation capability.
- Compared results with three QUATRE variants, two DE variants, and two PSO variants on testing CEC2013 benchmark is to evaluate confirming the performance of the proposed algorithm.
- An applied AMG-QUATRE to the node localization in WSN by modifying the average hop distance improving the DV-Hop algorithm and implementing the proposed algorithm to obtain the position of the nodes in WSN.

The remainder of the paper is organized as follows. Section 2 reviews the original QUATRE algorithm and the localization in WSN problem. Section 3 presents the proposed AMG-QUATRE algorithm and its application to node localization in WSN. Section 4 analyses the experimental results of the proposed algorithm for CEC2013 benchmark set with 28 benchmark functions and simulation results for node localization in WSN. Finally, the conclusion is given in Section 5.

## 2. Related Works

### 2.1. Original QUATRE Algorithm

QUATRE [15] is a swarm-based algorithm for solving single-objective optimization problems over continuous space. QUATRE is a combination of the acronym quasi-affine transformation evolution. Individuals in this algorithm evolve with a quasi-affine transformation form, which is extended from the affine transformation in geometry from one affine space to another. The exact evolution Equation for QUATRE is shown as follows.

(1) $$X\leftarrow M⊗X+\bar{M}⊗B$$

X represents the target population matrix consisting of *ps* target individuals, $X={\left[X_{1,G},X_{2,G},\ldots ,X_{ps,G}\right]}^{T}$. $X_{i,G}=[x_{i1},x_{i2},\ldots ,x_{iD}]$, i∈{1,2,…,ps}, is the ith row vector of the X, which represents the position of ith individual at the generation Gth, and it is a candidate solution to the optimization problem, and D represents the dimension of problem. B denotes the mutation/donor matrix, $B={\left[B_{1,G},B_{2,G},\ldots ,B_{ps,G}\right]}^{T}$. ⊗ represents component-wise multiplication of the corresponding elements in the two matrices. M is an evolution matrix, and $\bar{M}$ represents a binary operation of inverting all the elements in M. All elements of these two matrices are binary values, either 1 or 0. The inverse values of one elements are zeros, and inverse values of zero elements are ones. Equation (1) implements an alternative method of crossover operation in the DE algorithm.

M is an automatically generated matrix which is transformed from an initial matrix $M_{init}$. For simplicity, $M_{init}$ is initialized according to a lower triangular matrix whose elements equaling to ones. Equation (2) shows an example of the initialization and transformation when the dimension number *D* equals to population size *ps*. There are two consecutive steps incorporated in transforming from $M_{init}$ to M, indicated by “~” operator in Equation (2). First, the elements in each D-dimension row vector of $M_{init}$ are randomly permuted. Second, the sequence of all the row vectors is randomly permuted with each row vector unchanged. When the population size *ps* is larger than the vector dimension, the initial matrix $M_{init}$ requires to be extended according to *ps*. Equation (3) gives an example of extension with ps=2D+2. More generally, when ps%D=k, the top *k* rows of the D×D lower triangular matrix are included in $M_{init}$, and M adaptively changed in accordance with the change of $M_{init}$ [15].

(2) $$M_{init}=\left[\begin{array}{cccc} 1 & & & \\ 1 & 1 & & \\ & & \ldots & \\ 1 & 1 & \ldots & 1 \end{array}\right]\sim \left[\begin{array}{cccc} 1 & 1 & \ldots & 1\\ & 1 & 1 & \\ & & \ldots & \\ & 1 & & \end{array}\right]=M$$

(3) $$M_{init}=\left[\begin{array}{cccc} 1 & & & \\ 1 & 1 & & \\ & & \ldots & \\ 1 & 1 & \ldots & 1\\ 1 & & & \\ 1 & 1 & & \\ & & \ldots & \\ 1 & 1 & \ldots & 1\\ & & \ldots & \\ 1 & & & \\ 1 & 1 & & \end{array}\right]\sim \left[\begin{array}{cccc} 1 & & \ldots & 1\\ & 1 & \ldots & \\ & \ldots & & \\ 1 & 1 & & \\ & 1 & & \\ 1 & \ldots & 1 & 1\\ & & 1 & \\ 1 & 1 & \ldots & 1\\ & & \ldots & \\ 1 & \ldots & 1 & 1\\ & & 1 & \end{array}\right]=M$$

There are several different mutation schemes [19,28] introduced for calculation B in QUATRE algorithm. The seven mutation schemes are listed in Table 1. $X_{gbest,G}={\left[X_{gbest,G},X_{gbest,G},\ldots ,X_{gbest,G}\right]}^{T}$ in Table 1 is the global best matrix at generation Gth with each row vector equaling to the global best individual $X_{gbest,G}$. $X_{ri,G}$, i∈{1,2,…,5}, in Table 1 is a random matrix which is generated by randomly permutating the sequence of row vectors in the target population matrix X at generation Gth. The control parameter *F*, so-called mutation scaling factor, is a positive number for scaling the difference matrix, whose recommended domain is (0,1] for most optimization problems.

### 2.2. Statement of the Location Problem

In some applications of WSN, node locations play essential roles in its successful implementation, e.g., fire monitoring system, battlefield monitoring, animal monitoring, web tracking [29,30,31]. Practical applications of WSN are challenging in design under constraints such as limited sensor power and low computational cost [32,33]. A suitable localization algorithm appreciates in developing application WSN. DV-Hop node localization has been widely used in many application because of its simplicity, easy implementing. However, the DV-Hop method has its accuracy still low. So, there are many improvements in the original DV-Hop algorithm have been proposed in the literature that attempts reducing its estimation error. The improved DV-Hop algorithms often have features of given anchor nodes, calculate hop distances, and estimate unknown nodes. Some approaches to improving localization algorithm included applying metaheuristics algorithms such as genetic algorithm (GA) for the DV-Hop and area coverage [34,35], PSO improved localization [36], differential evolution (DE) for localization [37], Improved DV-Hop algorithm based on teaching learning-based optimization (TLBO) [38], multi-objective firefly algorithm (MOFA) for node localization [39], shuffled frog leaping algorithm (SFLA) node localization, elephant herding optimization (EHO) for localization [40], elephant herding optimization (EHO) and tree growth algorithm adapted (TGA) for node localization [41]. There are three typical phases in the node localizations as follows.

In the first phase, every anchor node broadcasts a beacon packet $\left\{id,x_{i},y_{i},hop_{i}\right\}$, including its identifier id, location coordinate $\left(x_{i},y_{i}\right)$ and a zero-initialized hop-count value $hop_{i}$, to its neighbor nodes. Each node maintains its own hop-count table, which contains ids, location coordinates and minimum hop-count values of all anchor nodes. When any node receives a packet, it first checks if there is a record for the corresponding anchor node in the table. If the record does not exist, the anchor node is recorded in the table of this node. If the record exists and the hop-count value in the packet is less than the value recorded in the table, the value of the record will be updated. Then, the hop-count value is increased by one to form a new packet. This new packet will be sent to its neighbor nodes. Otherwise, if the hop-count value in the packet is greater than or equal to the value recorded in the table, the received packet is ignored. By this process, all nodes in the entire network get minimum hop-count value from every anchor node.

In the second phase, we estimate the distance between anchor node and the unknown node. Firstly, every anchor node estimates the average hop distances (hop size) using Equation (4):

(4) $$Hopsize_{i}=\frac{\sum_{j=1,j\neq i}^{m}\sqrt{{\left(x_{i}-x_{j}\right)}^{2}+{\left(y_{i}-y_{j}\right)}^{2}}}{\sum_{j=1,j\neq i}^{m}hop_{ij}}$$

where *i* is the id of anchor node *i*, *m* is the number of anchor nodes, $hop_{ij}$ is the minimum hop-count value between anchor nodes *i* and *j*, $\left(x_{i},y_{i}\right)$, $\left(x_{j},y_{j}\right)$ are the position coordinates of anchor nodes *i* and *j*. Then, each anchor node broadcasts one more packet containing its $Hopsize_{i}$ in the entire network by using controlled flooding. The unknown node will store the first hop-size, which it receives and transmits the hop-size packet to neighbor nodes. Next, each unknown node calculates the distances to all anchor nodes using Equation (5) which is given as follow.

(5) $$d_{uv}=hopsize_{i}\times hop_{uv}$$

where $hopsize_{i}$ is the average hop distances (hop size) obtained by unknown node *u* from nearest anchor node *i*, $hop_{uv}$ is the minimum hop-count value between the unknown node *u* and the anchor node *v*.

In the last phase, we estimate the coordinate of each unknown node by using the multilateration method. Suppose the (x,y) denotes the position coordinate of unknown node *U*,$\left(x_{i},y_{i}\right)$ denotes position coordinate of *i*th anchor node, $d_{i}$, i=1…m, represent the distance between the unknown node *U* and all anchor nodes. Then, we can obtain the following Equation (6).

(6) $$\{\begin{array}{c} {\left(x_{1}-x\right)}^{2}+{\left(y_{1}-y\right)}^{2}=d_{1}^{2}\\ {\left(x_{2}-x\right)}^{2}+{\left(y_{2}-y\right)}^{2}=d_{2}^{2}\\ \vdots \\ {\left(x_{m}-x\right)}^{2}+{\left(y_{m}-y\right)}^{2}=d_{2}^{2} \end{array}$$

Equation (7) can be obtained by expanding Equation (6) and subtract the first (*m* − 1) as the equations follows.

(7) $$\{\begin{array}{l} x_{1}^{2}-x_{m}^{2}+2(x_{1}-x_{m})x+y_{1}^{2}-y_{m}^{2}-2(y_{1}-y_{m})y=d_{1}^{2}-d_{n}^{2}\\ x_{2}^{2}-x_{m}^{2}+2(x_{2}-x_{m})x+y_{2}^{2}-y_{m}^{2}-2(y_{2}-y_{m})y=d_{2}^{2}-d_{n}^{2}\\ \vdots \\ x_{m-2}^{2}-x_{m}^{2}+2(x_{m-2}-x_{m})x+y_{m-2}^{2}-y_{m}^{2}-2(y_{m-2}-y_{m})y=d_{m-2}^{2}-d_{n}^{2}\\ x_{m-1}^{2}-x_{m}^{2}+2(x_{m-1}-x_{m})x+y_{m-1}^{2}-y_{m}^{2}-2(y_{m-1}-y_{m})y=d_{m-1}^{2}-d_{n}^{2} \end{array}$$

Equation (7) can be written in matrix form of AX=B, where A, X, and B are given by the following Equations.

(8) $$X=\left[\begin{array}{l} x\\ y \end{array}\right]$$

(9) $$A=\left[\begin{array}{l} 2(x_{1}-x_{m})\\ 2(x_{2}-x_{m})\\ \vdots \\ 2(x_{2}-x_{m}) \end{array}\begin{array}{l} 2(y_{1}-y_{m})\\ 2(y_{2}-y_{m})\\ 2(y_{m-1}-y_{m}) \end{array}\right]$$

(10) $$B=\left[\begin{array}{l} x_{1}^{2}+y_{1}^{2}-x_{m}^{2}-y_{m}^{2}+d_{m}^{2}-d_{1}^{2}\\ x_{2}^{2}+y_{2}^{2}-x_{m}^{2}-y_{m}^{2}+d_{m}^{2}-d_{2}^{2}\\ \vdots \\ x_{m-1}^{2}+y_{m-1}^{2}-x_{m}^{2}-y_{m}^{2}+d_{m}^{2}-d_{m-1}^{2} \end{array}\right]$$

Coordinates of the unknown nodes U are obtained by using least-squares method with Equation (11).

(11) $$X=(A^{T}A{)}^{-1}A^{T}B$$

## 3. Adaptation Multi-Group QUATRE Algorithm and Its Application to Node Localization in WSN

### 3.1. Adaptation Multi-Group QUATRE Algorithm (AMG-QUATRE)

**Figure 1:**
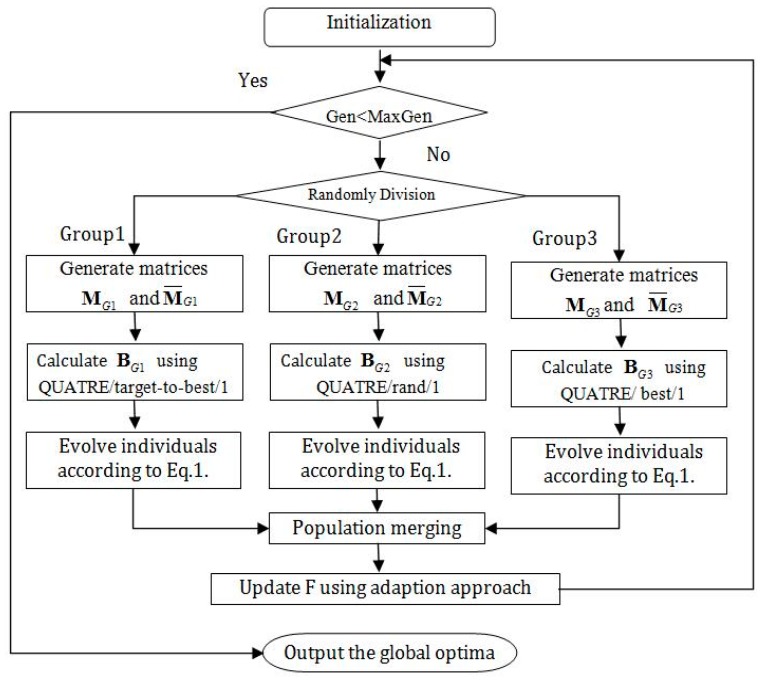
The main framework of adaptation multi-group quasi-affine transformation evolutionary algorithm (AMG-QUATRE).

The main idea of the proposed AMG-QUATRE algorithm is described in this section. As mentioned above, the canonical QUATRE algorithm has the problem of easily trapping into local optima and premature convergence. In order to reduce above weaknesses, we proposed an improved QUATRE, called AMG-QUATRE, which is made up of population initialization, random population division, group evolution, and group recombining and adaption method for updating parameter scale factor *F*. Figure 1 shows an illustration of the main framework of proposed AMG-QUATRE algorithm.

#### 3.1.1. Population Division and Mutation Schemes

QUATRE variants adopting different mutation schemes usually have different performance in solving different optimization problems, due to different mutation schemes having different exploration and exploitation capability [15]. The mutation scheme “QUATRE/best/1” employs the best individual found so far to guide the search direction, so it has very fast convergence speed and excellent local search capability around the best individual. This mutation scheme performs well when solving unimodal problems. But it is more likely to fall into local optimum and thereby lead to a premature convergence when solving multimodal problems. The mutation scheme “QUATRE/rand/1” is the most commonly used, powerful and robust scheme. Although it has slow convergence speed, it has stronger exploration capability to maintain population diversities. This mutation scheme is usually more suitable for solving multimodal problems. The mutation scheme “QUATRE/target-to-best/1” has good exploration and convergence capabilities, because the individuals using this scheme are guided by the best individual found so far and two randomly selected individuals. To make full use these different schemes, in our proposed algorithm, we utilize multi-group approach to incorporating their advantages. As can be seen from Figure 1, in our proposed algorithm, we first initialize the individual population and then randomly divide the population into three groups, say group1, group2 and group3, respectively. And each group use different mutation scheme, group1 uses the scheme “QUATRE/target-to-best/1”, group2 employs the scheme “QUATRE/rand/1”, and group3 utilizes the scheme “QUATRE/best/1”, respectively. Therefore, this multi-group partition and different group adopting different mutation scheme approach can be traded off between the population diversity and convergence speed.

#### 3.1.2. Adaptation Scale Factor

In the whole evolution phases, scale factor plays an important role in affecting the exploration and development capability of QUATRE algorithm. In [15], Meng et al. illustrate that QUATRE with different scaling factor values is suitable for different optimization problems. And there is no fixed parameter setting to obtain the optimal performance for all types of problems. Three categories parameter control methods have been proposed to adjust the evolutionary algorithms parameter values dynamically [27]. Nowadays, most of researchers focused on the adaptive control parameters. Hence, we use the adaptation scheme [22] to dynamically update the value of scale factor. In the proposed algorithm, scale factor *F* obeys Cauchy distribution, $F\sim C(\mu_{F},\sigma_{F})$, with location parameter $\mu_{F}$ and scale parameter $\sigma_{F}$. The initial value for $\mu_{F}$ is set to 0.5, and $\sigma_{F}$ is set to a constant value 0.1 during the whole search process. Each individual in the population has its own F value which can be updated according to Equation (12).

(12) $$F_{i}=C_{i}(\mu_{F},\sigma_{F})$$

In each generation $\mu_{F}$ is updated by weighted Lehmer mean according to Equation (13). $S_{F}$ indicates the set of parameters $F_{i}$ with which the associated individuals can obtain a better fitness value. $f(U_{i,G})$ represents the fitness value for trial vector of the *i*th individual at generation *G*, and $f(U_{i,G})$ represents the fitness value for target vector of the *i*th individual at generation *G*. $\Delta f_{i}$ represents the corresponding fitness difference of the *i*th individual with the associated parameter $F_{i}$.

(13) $$\{\begin{array}{l} w_{F_{i}}=\frac{\Delta f_{i}}{\sum_{F_{i}\in S_{F}}\Delta f_{i}}\\ \Delta f_{i}=f(U_{i,G})-f(X_{i,G})\\ \mu_{F}=\frac{\sum_{F_{i}\in S_{F}}w_{F_{i}}.F_{i}^{2}}{\sum_{F_{i}\in S_{F}}w_{F_{i}}.F_{i}} \end{array}$$

**Algorithm 1. Shows the Pseudo Code of AMG-QUATRE Algorithm.**

**  // Initialization phase**
  Initialize the searching space V, dimension D, Set the generation counter Gen=1, randomly initialize the population X with ps individuals, and evaluate fitness values of all individuals, set initial $\mu_{F}=0.5$, $\sigma_{F}=0.1$.  **// Main loop**
  1: **while** stopping criterion is not satisfied **do**
  2:  Randomly partition the population into three groups, group1, group2 and group3
  3:  Generate matrices $M_{group1}$ and ${\bar{M}}_{group1}$, $M_{group2}$ and ${\bar{M}}_{group2}$, $M_{group3}$ and ${\bar{M}}_{group3}$, using Equation (3).  4:  Calculate mutation matrix $B_{group1}$ using QUATRE/target-to-best/1, $B_{group2}$ using QUATRE/rand/1, $B_{group3}$ using QUATRE/best/1.  5:  Evolve individuals in each group using Equation (1).  6:  Evaluate fitness values of all individuals.  7:   **for**
i=1;i≤ps;i++
**do**
  8:    **if**
$f(X_{i})\leq f(X_{pbest,i})$
**then**
  9:     $X_{pbest,i}=X_{i}$
  10:    **end if**
  11:   **end for**
  12:  $X=X_{pbest}$, $X_{gbest}=opt\{X_{pbest}\}$.  13:  Update scale factor F according to Equation (12).  14:  Gen=Gen+1
  15: **end while**
  **Output:** The global optimum $X_{gbest}$, global best fitness value $f(X_{pbset})$.

### 3.2. Our Proposed Algorithm for DV-Hop Localization Algorithm

In this subsection, we introduce the proposed AMG-QUATRE algorithm for the DV-Hop node localization in WSN. As we all know, the estimated distance between nodes is obtained multiplying the hop-count values by the hop size of the anchor node. However, when the number of hops between the anchor node and the unknown node is greater than two, this method suffers from a problem of poor distance estimation that leads decreasing accuracy of localization. The main aim of the localization problem in WSN is to minimize the estimation error and to improve the localization accuracy. In order to reduce the estimation error, we present an improved DV-Hop based on AMG-QUATRE for node localization in WSN. The proposed algorithm first refines the hop size of anchor nodes according to the least square error criterion, and then calculates the hop size of unknown nodes by weighting the received hop size from all anchor nodes. Finally, the proposed algorithm employs AMG-QUATRE algorithm to estimate the locations of unknown nodes.

#### 3.2.1. Modification of Hop Size

The hop size of anchor nodes mainly determines the accuracy of the estimated distance. Localization accuracy could be improved by modifying the hop size of anchor nodes according to the references [42]. The least square error criterion is used to refine the average hop distance of each anchor node as follow.

(14) $$Hopsize_{i}=\frac{\sum_{j=1,j\neq i}^{m}hop_{ij}d_{ij}}{\sum_{j=1,j\neq i}^{m}hop_{ij}^{2}}$$

where *d_ij_* represents the distance from anchor node *i* to anchor node *j*. Then, the hop size of unknown nodes is calculated by weighting the *m* received hop size from all anchor nodes using Equations (15) and (16).

(15) $$Hopsize_{u}=\sum_{i=1}^{m}w_{i}Hopsize_{i}$$

(16) $$w_{i}=\frac{hop_{i}}{\sum_{j=1}^{m}hop_{j}}$$

where $hop_{i}$ represents the hop-count value from anchor node *i* to unknown node *u* and *m* is the number of all anchor nodes.

#### 3.2.2. Using AMG-QUATRE Algorithm to Locate the Unknown Nodes

In this subsection, the proposed AMG-QUATRE algorithm is applied to minimize squared error of the estimated distances that means to locate unknown nodes. The second phase of DV- Hop algorithm, the distance between the unknown node and anchor node $d_{ui}$ is an estimated value, so it may be error prone. Let $\left(x^{\prime },y^{\prime }\right)$ is the actual position coordinate of unknown node, $\left(x_{i},y_{i}\right)$ denotes position coordinate of *i*th anchor node. The squared error of the estimated distances is defined as follows.

(17) $$e={\sum_{i=1}^{m}\left(\sqrt{{\left(x^{\prime }-x_{i}\right)}^{2}-{\left(y^{\prime }-y_{i}\right)}^{2}}-d_{ui}\right)}^{2}$$

The main aim of the localization problem in WSN is to minimize this error. Take into consideration that the distance estimation error also increases as the number of hops increases. So the Equation (17) can be deal in a weighted way by the reciprocal of hop-count. Hence, the fitness function of the AMG-QUATRE can be defined as follows.

(18) $$f(x^{\prime },y^{\prime })=\min({\sum_{i=1}^{m}{\left(\frac{1}{hop_{ui}}\right)}^{2}(\sqrt{{\left(x^{\prime }-x_{i}\right)}^{2}-{\left(y^{\prime }-y_{i}\right)}^{2}}-d_{ui})}^{2})$$

For every unknown node, we use an independent AMG-QUATRE optimization process to reckon its location. The individual encoding of AMG-QUATRE for location estimation is two dimension variables $\left(x_{i},y_{i}\right)$ representing the coordinate of unknown node. The optimization process contains four steps as follows.

Step 1, initialize parameters of AMG-QUATRE and ps individuals.
Step 2, generate donor matrices.
Step 3, generate trial matrices.
Step 4, select a better vector between the trial vector and its corresponding target vector to enter the next generation. Repeat the above steps 2 to 4 until the stop condition is satisfied.

**Figure 2:**
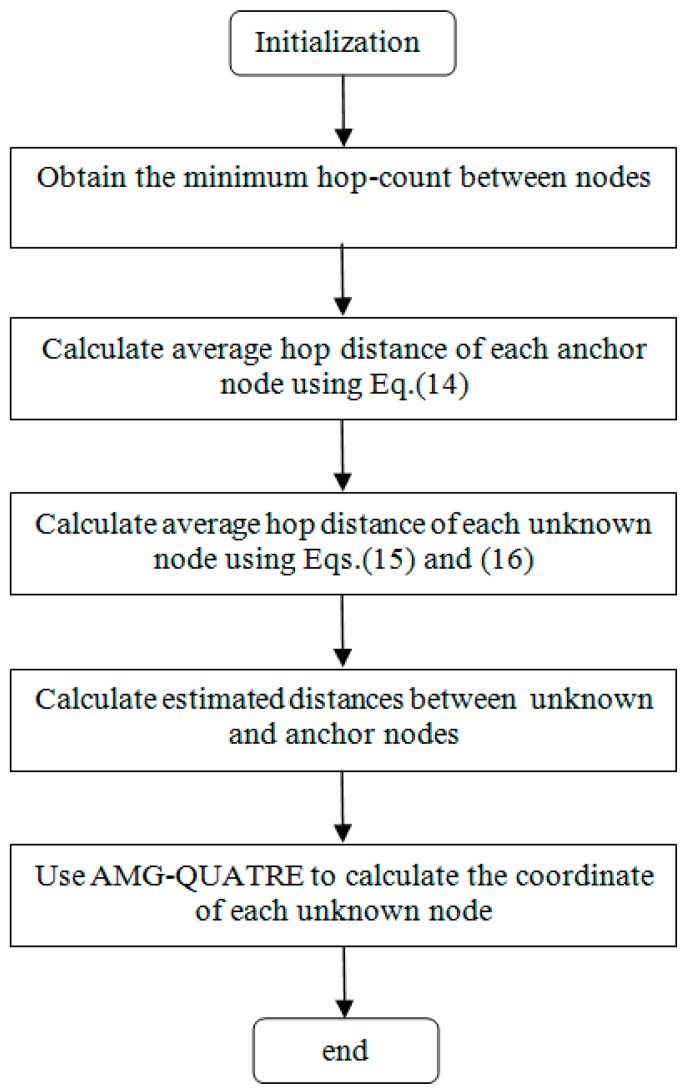
The flowchart of the proposed distance vector-hop (DV-Hop) algorithm based on AMG-QUATRE.

The whole flowchart of the proposed DV-Hop algorithm based on AMG-QUATRE is given in Figure 2.

## 4. Experimental Analysis

In this section, two groups of experiments are conducted to verify the performance of our proposed AMG-QUATRE algorithm and its application to node localization in WSN. 

### 4.1. Simulation Results on Standard Bound-Constrained Benchmarks

A benchmark set of CEC2013 [43] is utilized to verify the performance of our proposed AMG-QUATRE algorithm in the following experiment. 

CEC2013 has 28 benchmark functions, including five (*f*_1_–*f*_5_) unimodal functions, fifteen (*f*_6_–*f*_20_) multi-modal functions and eight (*f*_21_–*f*_28_) composition functions. These benchmark functions’ definition can be found in [43]. All these benchmark functions are shifted to the same global minimum $O\left\{o_{1},o_{2},\ldots ,\;o_{d}\right\}$.

**Figure 3:**
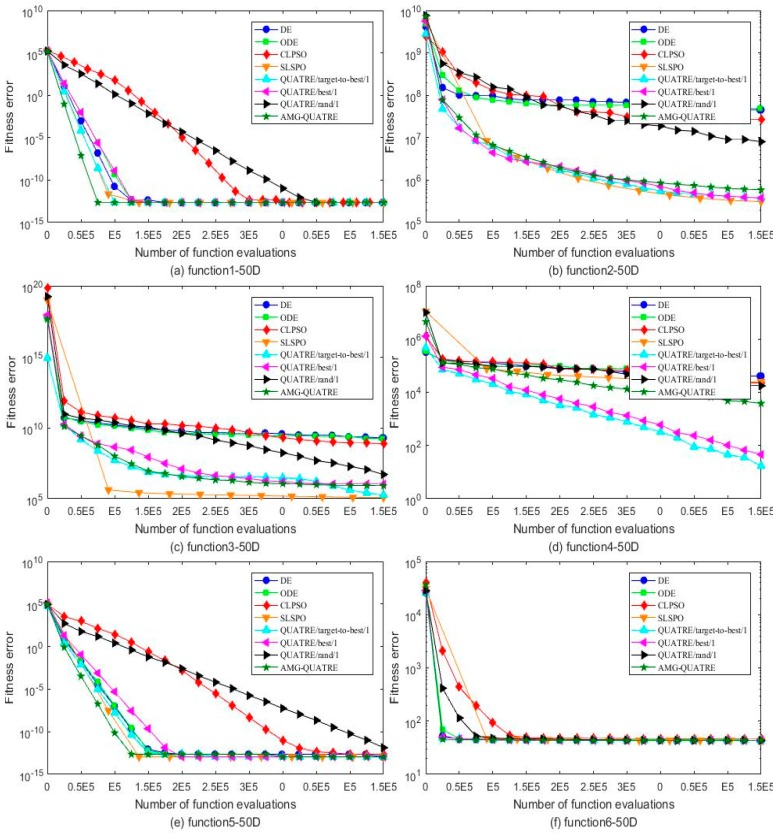
Comparison of the best of fitness errors for functions *f*_1_–*f*_6_ with 50D optimization. (**a**) $f_{1}$; (**b**) $f_{2}$; (**c**)$f_{3}$; (**d**)$f_{4}$; (**e**) $f_{5}$; (**f**) $f_{6}$.

**Figure 4:**
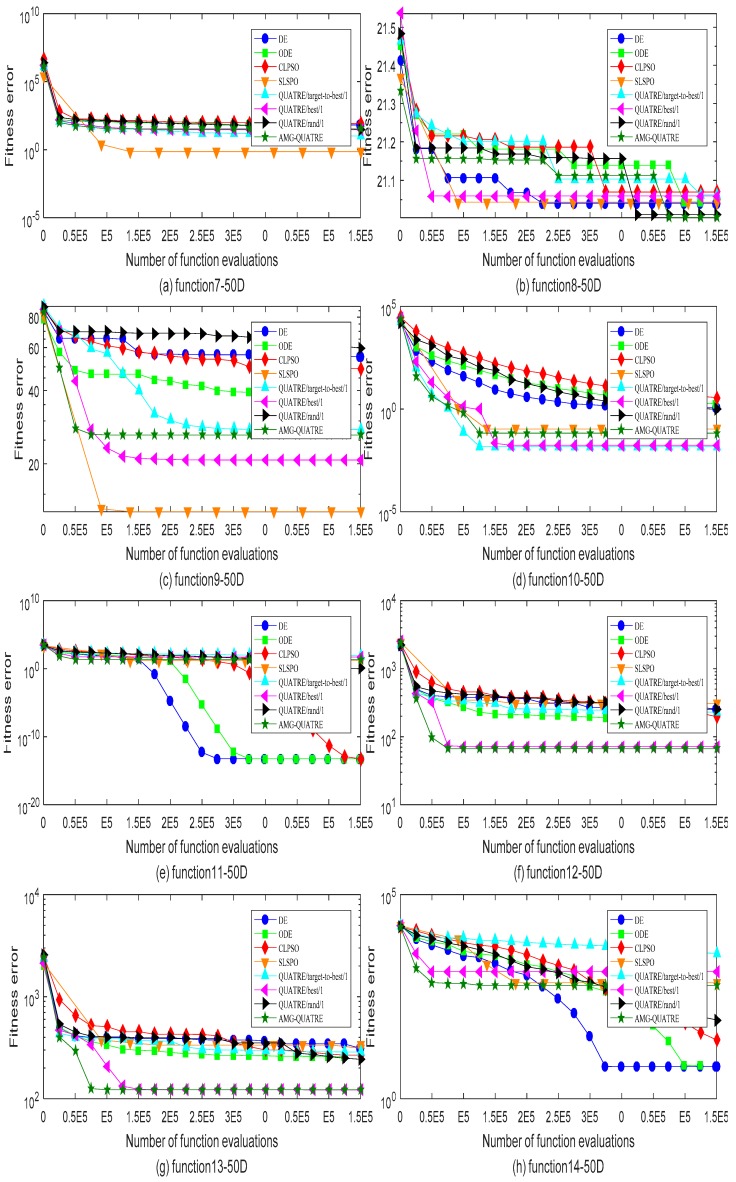
Comparison of the best of fitness errors for functions *f*_7_–*f*_14_ with 50D optimization. (**a**) $f_{7}$; (**b**) $f_{8}$; (**c**)$f_{9}$; (**d**)$f_{10}$; (**e**) $f_{11}$; (**f**)$f_{12}$; (**g**) $f_{13}$; (**h**)$f_{14}$.

**Figure 5:**
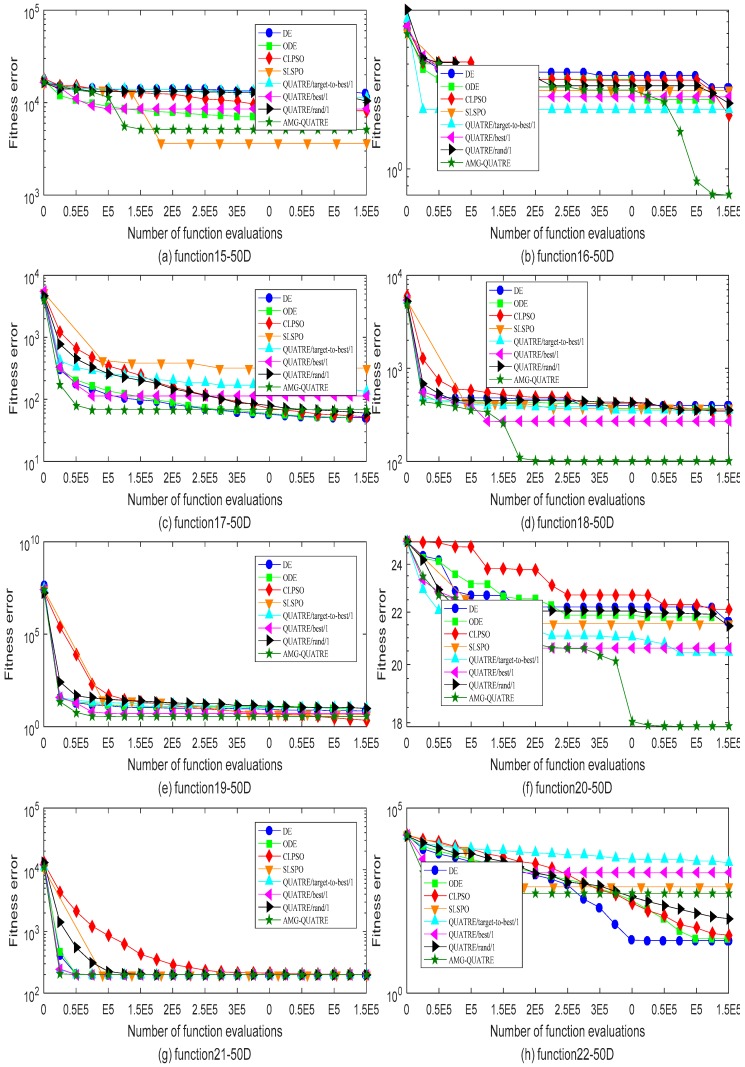
Comparison of the best of fitness errors for functions *f*_15_–*f*_22_ with 50D optimization. (**a**) $f_{15}$; (**b**) $f_{16}$; (**c**)$f_{17}$; (**d**)$f_{18}$; (**e**) $f_{19}$; (**f**)$f_{20}$; (**g**) $f_{21}$; (**h**) $f_{22}$.

**Figure 6:**
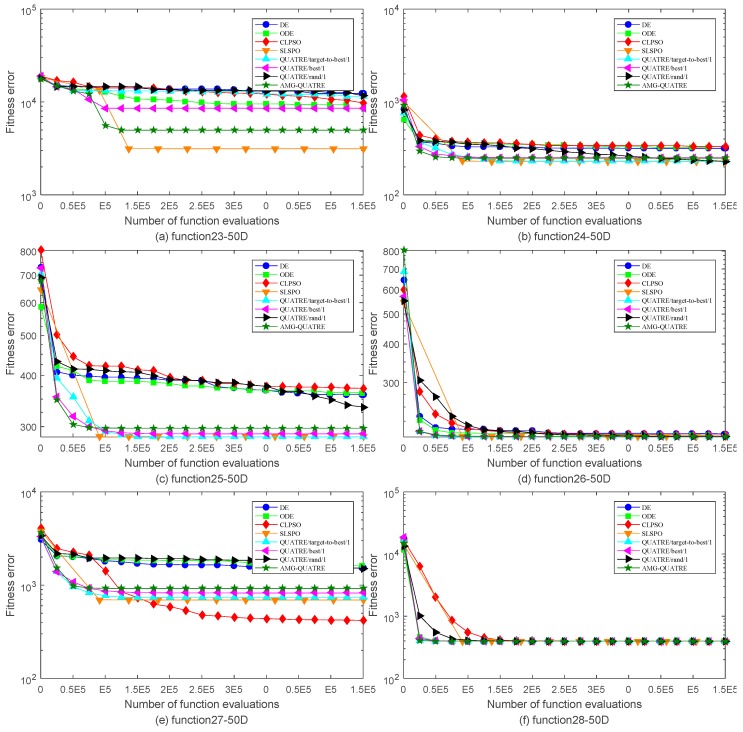
Comparison of the best of fitness errors for functions *f*_23_–*f*_28_ with 50D optimization. (**a**) $f_{23}$; (**b**) $f_{24}$; (**c**)$f_{25}$; (**d**)$f_{26}$; (**e**) $f_{27}$; (**f**) $f_{28}$.

The proposed AMG-QUATRE is compared with the three QUATRE variants [15,19] “QUATRE/target-to-best/1”, “QUATRE/rand/1” and “QUATRE/best/1” because of AMG-QUATRE utilizing these three mutation schemes. We also compare it with DE [8], ODE [44], CLPSO [45], and SLPSO [46] due to the QUATRE algorithm has relationship with PSO and DE algorithms as described in paper [15]. The parameters of these algorithms are set in Table 2 according to the recommended values of the referenced papers. The dimension number *D* of all functions is set to 50. The maximal number of function evaluation (NFE) is set to 10000×D. We run each of the contrasted algorithms on every benchmark function 20 times independently. Table 3 and Table 4 collect the best and mean/standard deviation of the function error $\Delta f=f_{i}-f_{i}^{*}$ respectively. Symbols “+”, “−” and “=” in parentheses after the values “Best” and “Mean/ standard deviation” means “better performance”, “worse performance” and “similar performance” respectively. Wilcoxon’s signed rank test with a level of significance α=0.05 is used for the evaluation of “Mean/Std”. “Best” are measured by their associated arithmetic values, and we use the criterion “The smaller the better”. The simulation results of all benchmark functions are plotted in Figure 3, Figure 4, Figure 5 and Figure 6.

As can see from Table 3, the AMG-QUATRE outperform the other seven contrasted algorithms significantly over 28 benchmark functions from the optimization accuracy perspective. Comparing with DE algorithm, the proposed AMG-QUATRE algorithm obtained 20 better performances, 4 similar performances, and 4 worse performances out of 28 benchmarks. Comparing with ODE algorithm, it obtained 20 better performances, 4 similar performances, and 4 worse performances out of 28 benchmarks. Comparing with CLPSO algorithm, it obtained 20 better performances, 2 similar performances, and 6 worse performances out of 28 benchmarks. Comparing with SLPSO algorithm, it obtained 12 better performances, 5 similar performances, and 11 worse performances out of 28 benchmarks. Comparing with “QUATRE/best/1”, it obtained 14 better performances, 4 similar performances, and 10 worse performances out of 28 benchmarks. Comparing with “QUATRE/rand/1”, it obtained 19 better performances, 2 similar performances, and 7 worse performances out of 28 benchmarks. Comparing with “QUATRE/target-to-best/1”, it obtained 14 better performances, 5 similar performances, and 9 worse performances out of 28 benchmarks. The convergence speeds of these algorithms are contrasted by plotting the convergence curves of best values in Figure 3, Figure 4, Figure 5 and Figure 6. As can see from the figures, the proposed AMG-QUATRE algorithm performs best on $f_{8}$, $f_{12}$, $f_{13}$, $f_{16}$, $f_{18}$, $f_{20}$ than other competing algorithms.

From Table 4, we can see that, for the “Mean/standard deviation” value, the proposed AMG-QUATRE algorithm obtained 18 better performances, 5 similar performances, and 5 worse performances out of 28 benchmarks by comparing with DE algorithm. It obtained 18 better performances, 6 similar performances, and 4 worse performances by comparing with ODE algorithm. It obtained 18 better performances, 3 similar performances, and 7 worse performances by comparing with CLPSO algorithm. It obtained 13 better performances, 6 similar performances, and 9 worse performances by comparing with SLPSO algorithm. It obtained 13 better performances, 11 similar performances, and 4 worse performances by comparing with “QUATRE/best/1” algorithm. It obtained 16 better performances, 4 similar performances, and 8 worse performances by comparing with “QUATRE/rand/1” algorithm. It obtained 13 better performances, 6 similar performances, and 9 worse performances by comparing with “QUATRE/target-to-best/1” algorithm. Overall, our proposed AMG-QUATRE algorithm performed better than the other seven competing algorithms.

### 4.2. Simulation Results of Applied AMG-QUATRE to Node Localization in WSN

Simulations were conducted to verify the performance of our proposed DV-Hop based on AMG-QUATRE. We compared simulation results of our proposed algorithm with the standard DV-Hop, Hyperbolic-DV-hop, DV-Hop based on PSO, and DV-Hop based on DE. Hyperbolic-DV-hop is the algorithm that uses 2D Hyperbolic to replace the multilateration method in original DV-Hop. Using 2D Hyperbolic to estimate the location of unknown node can be found in references [42,47]. These five algorithms were implemented by MATLAB 2016a. The final performance of all the experimental results are the average of 20 runs of independent experiments. In simulations, sensor nodes were randomly scattered in 2-dimensional fixed square region of 100 m × 100 m. The parameter settings of simulation are shown in Table 5. The other parameters of AMG-QUATRE and DE are the same as Section 4.1. The parameters of PSO are $c_{1}=c_{2}=2.05,w_{\max}=0.9,\;\mathrm{and}\;w_{\min}=0.4$.

The average localization error is considered as performance metrics, which is given as follows.

(19) $$average\;localization\;error=\frac{\sum_{i=1}^{n}\sqrt{{\left(x_{i}-x_{i}^{\prime }\right)}^{2}+{\left(y_{i}-y_{i}^{\prime }\right)}^{2}}}{n\times R}$$

where *n* is the total number of unknown nodes, $\left(x_{i},y_{i}\right)$ is the estimated coordinate of unknown node and $\left(x_{i}^{\prime },y_{i}^{\prime }\right)$ is the actual coordinates and *R* is the communication range of sensors.

#### 4.2.1. Sensitivity of Varying Anchor Node Ratio

**Figure 7:**
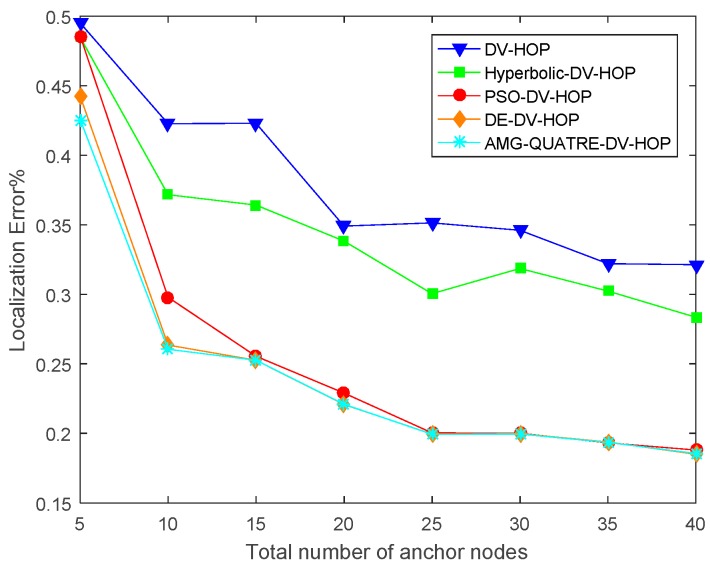
Comparison of location error of the applied AMG-QUATRE with the other methods for different anchor nodes.

**Figure 8:**
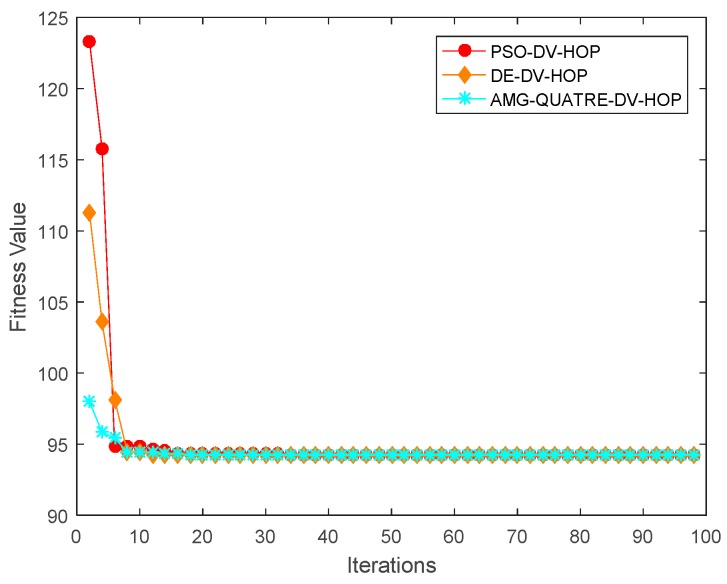
Comparison of convergence curve of the applied AMG-QUATRE with particle swarm optimization (PSO) and differential evolution (DE) methods for single simulation (Number of sensor node 200, Number of anchor node 40, Communication range 20).

In this experiment, there are 200 sensor nodes, each with a communication range of 20 m, randomly scattered in a sensing region of 100 m × 100 m. The number of anchor nodes gradually varies from 5 to 40. The experimental results are shown in Figure 7 and Table 6. Convergence curve for metaheuristics of one single simulation run is shown in Figure 8.

We can see from Figure 7 and Table 6 that our proposed algorithm achieved better performance than the DV-Hop, Hyperbolic-DV-hop and PSO-DV-hop algorithms. Our proposed algorithm had similar a performance with the DE-DV-hop algorithm. The average localization error of the proposed algorithm is about 13.6%, 10.3% and 1.4% lower than the standard DV-Hop algorithm, Hyperbolic-DV-hop algorithm and PSO-DV-hop algorithm, respectively. It is also observed from Figure 7 that as the number of anchor node increased, the average localization error of all algorithms also decreased. This is because as the number of anchor nodes increases, the average hop distances of anchor nodes become more accurate and the errors of the estimated distances become smaller.

#### 4.2.2. Sensitivity of Varying Communication Range

**Figure 9:**
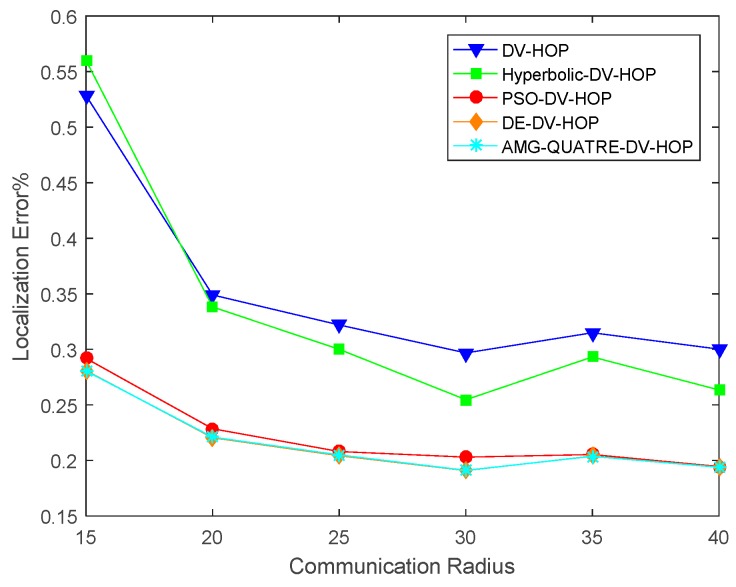
Comparison of location errors of the applied AMG-QUATRE with the other methods for a different communication range.

**Figure 10:**
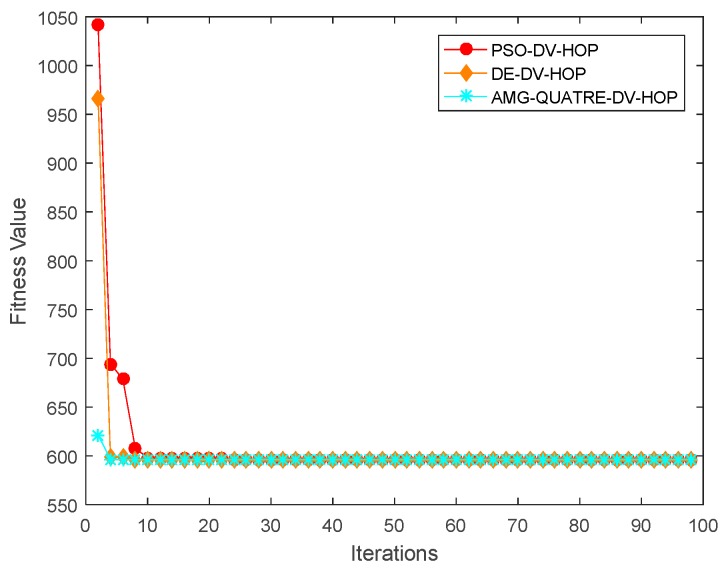
Comparison of convergence curve of the applied AMG-QUATRE with PSO and DE methods for single simulation (Number of sensor node 200, Number of anchor node 20, Communication range 40).

In this experiment, there were 200 sensor nodes with 10% anchor nodes randomly scattered in a sensing region of 100 m × 100 m. The communication range varies from 15 to 40 m. The experimental results are shown in Figure 9 and Table 7. Convergence curve for metaheuristics of one single simulation run is shown in Figure 10.

We can see from Figure 9 and Table 7 that our proposed algorithm achieved better performance than the DV-Hop, Hyperbolic-DV-hop and PSO-DV-hop algorithms. Our proposed algorithm had a similar performance with the DE-DV-hop algorithm. The average localization error of our proposed algorithm was about 13.6%, 11.9% and 0.6% lower than the standard DV-Hop algorithm, Hyperbolic-DV-hop algorithm and PSO-DV-hop algorithm, respectively. It was also observed that when the communication range exceeds 25m, the average localization error changes very little. This is because with the increase of communication range, the hop-count between sensor nodes decreases and hop size of anchor nodes increases.

#### 4.2.3. Sensitivity of Node Density

**Figure 11:**
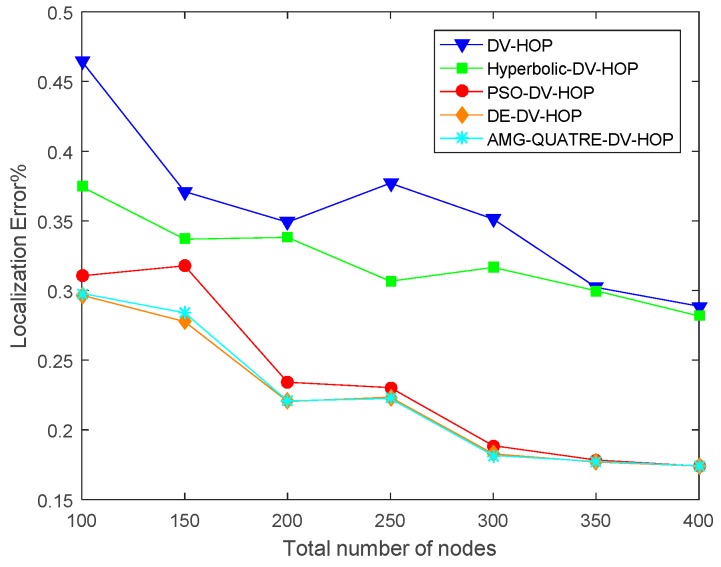
Comparison of average localization error of the applied AMG-QUATRE with the other methods for the different number of sensor nodes.

**Figure 12:**
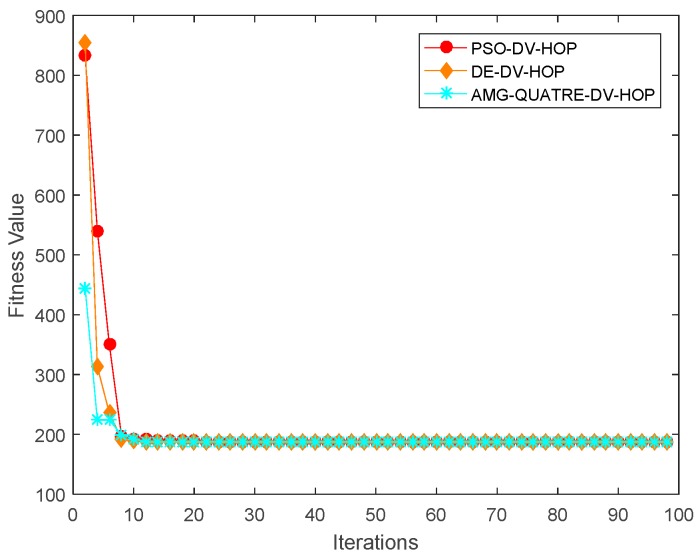
Comparison of convergence curve of the applied AMG-QUATRE with PSO and DE methods for single simulation (Number of the sensor node 400, Number of anchor node 40, Communication range 20).

In this simulation experiment, the number of sensor nodes varied from 100 to 400 with 10% anchor nodes randomly scattered in a 100 m × 100 m sensing region. The communication range of each sensor node is set to 20 m. The experimental results are shown in Figure 11 and Table 8. Convergence curve for metaheuristics of one single simulation run is shown in Figure 12.

We can see from Figure 11 and Table 8 that our proposed algorithm achieved better performance than the DV-Hop, Hyperbolic-DV-hop and PSO-DV-hop algorithms. Our proposed algorithm has similar performance with DE-DV-hop algorithm. The average localization error of our proposed algorithm was about 13.5%, 9.9% and 1.1% lower than the standard DV-Hop algorithm, Hyperbolic-DV-hop algorithm and PSO-DV-hop algorithm, respectively. It can be observed from Figure 11 that as the number of sensor nodes increased, the average localization error of all algorithms also decreased.

**Figure 13:**
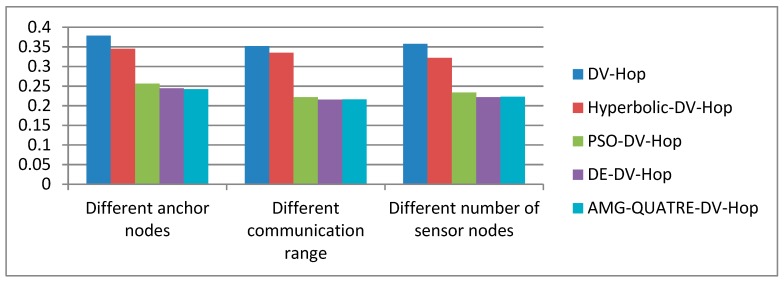
Comparison of average localization error of the applied AMG-QUATRE with the other methods.

Figure 13 shows a summary of the average localization error for the three groups of experiments including the rate of anchor nodes, the communicating ranges, and the density of nodes. It can be seen from Figure 13 that the proposed algorithm achieved a better performance than the DV-Hop, Hyperbolic-DV-hop, PSO-DV-hop algorithms. However, it is similar to the DE-DV-hop algorithm.

## 5. Conclusions

In this paper, a novel adaptation multi-group QUATRE algorithm (AMG-QUATRE) was presented for the global optimization problems. In AMG-QUATRE, the population has randomly partitioned into several groups. Each group adopted a different mutation scheme to reserve population diversity and improve the efficiency of the algorithm. Also, the control parameter scale factor *F* of mutation was updated adaptively during the search process to make a trade-off between exploration and exploitation ability. The CEC2013 test suite was used to verify the performance of the proposed algorithm. The compared experimental results with the other algorithms in the literature demonstrate that the proposed AMG-QUATRE algorithm not only has better performance than three QUATRE variants but also has better performance than two DE variants and two PSO variants in terms of converging rates and quality performance. 

The proposed AMG-QUATRE algorithm was also applied to enhance the node location accuracy of DV-Hop algorithm. In this application, we first refined the hop size of anchor nodes and then used AMG-QUATRE algorithm to estimate the locations of unknown nodes. We conducted several empirical scenarios of experiments such as on the different ratio of anchor nodes, diverse communication range, and different sizes of sensor networks. Simulated experimental results demonstrated that our proposed algorithm AMG-QUATRE-DV-hop achieves higher accuracy than the DV-Hop, Hyperbolic-DV-hop, and PSO-DV-hop algorithms. 

In future work, we will further expand the adopted efficient schemes to improve the performance of the evolution and swarm algorithms [48,49]. We will also apply the improved algorithms to different kinds of WSN problems, e.g., clustering approaches [50,51,52], hierarchical routing [53], deployment, and coverage in WSN [54].

## Figures and Tables

**Table 1:** The seven schemes of donor matrix B calculation.

No.	QUATRE/x/y	Equation
1	QUATRE/rand/1	$B=X_{r1,G}+F\cdot (X_{r2,G}-X_{r3,G})$
2	QUATRE/best/1	$B=X_{gbest,G}+F\cdot (X_{r1,G}-X_{r2,G})$
3	QUATRE/target/1	$B=X+F\cdot (X_{r1,G}-X_{r2,G})$
4	QUATRE/target-to-best/1	$B=X+F\cdot (X_{gbest,G}-X)+F\cdot (X_{r1,G}-X_{r2,G})$
5	QUATRE/rand/2	$B=X_{r1,G}+F\cdot (X_{r2,G}-X_{r3,G})+F\cdot (X_{r4,G}-X_{r5,G})$
6	QUATRE/best/2	$B=X_{gbest,G}+F\cdot (X_{r1,G}-X_{r2,G})+F\cdot (X_{r3,G}-X_{r4,G})$
7	QUATRE/target/2	$B=X+F\cdot (X_{r1,G}-X_{r2,G})+F\cdot (X_{r3,G}-X_{r4,G})$

**Table 2:** Parameters settings.

Algorithm	Parameters Settings
DE	F=0.5,Cr=0.1,ps=100
ODE	F=0.5,Cr=0.1,Jr=0.3,ps=100
CLPSO	iw∈[0.9,0.3],cc=1.49455,Pc∈[0,0.5],stay_num=7,v=rnd,vmax=0.2R
SLPSO	$M=100,c_{3}=0.005,PL\in [0,1]$
QUATRE variants	F=0.7,ps=100
AMG-QUATRE	$\mu_{F}=0.5,\sigma_{F}=0.1,ps=100$

**Table 3:** Comparison results of best value of 20-run fitness error among contrasted algorithms under CEC2013 test suite.

50D	DE/best/1/bin	ODE/best/1/bin	CLPSO	SLPSO	QUATRE/best	QUATRE/rand	QUATRE/target-to-best	AMP-QUATRE
1	2.273 × 10^−13^(=)	2.273 × 10^−13^(=)	2.2737 × 10^−13^(=)	2.2737 × 10^−13^(=)	0.0000 × 10^+00^(+)	0.0000 × 10^+00^(+)	2.2737 × 10^−13^(=)	2.2737 × 10^−13^
2	4.5454 × 10^+07^(−)	4.871 × 10^+07^(−)	5.9535 × 10^+05^(−)	3.1277 × 10^+05^(+)	3.8146 × 10^+05^(+)	8.0401 × 10^+06^(−)	3.7393 × 10^+05^(+)	5.8732 × 10^+05^
3	1.9098 × 10^+09^(−)	1.775 × 10^+09^(−)	8.0541 × 10^+06^(−)	1.2111 × 10^+05^(+)	1.0726 × 10^+06^(−)	5.1302 × 10^+06^(−)	1.7985 × 10^+05^(+)	8.3722 × 10^+05^
4	4.0671 × 10^+04^(−)	4.629 × 10^+04^(−)	3.818 × 10^+03^(−)	2.3913 × 10^+04^(−)	4.5832 × 10^+01^(+)	1.7578 × 10^+04^(−)	1.7273 × 10^+01^(+)	3.8289 × 10^+03^
5	1.1369 × 10^+1^ (=)	1.136 × 10^−13^(=)	1.1369 × 10^−13^(−)	1.1369 × 10^−13^(=)	1.1369 × 10^−13^(=)	1.3642 × 10^−12^(−)	1.1369 × 10^−13^(=)	1.1369 × 10^−13^
6	4.3447 × 10^+01^(−)	4.415 × 10^+01^(−)	4.3447 × 10^+01^(−)	4.3447 × 10^+01^(=)	4.3447 × 10^+01^(=)	4.3447 × 10^+01^(−)	4.3447 × 10^+01^(=)	4.3447 × 10^+01^
7	6.4767 × 10^+01^(−)	6.1526 × 10^+01^(−)	3.5117 × 10^+01^(−)	7.1569 × 10^−01^(+)	2.9215 × 10^+01^(+)	3.1212 × 10^+01^(+)	9.8822 × 10^+00^(+)	3.3071 × 10^+01^
8	2.1041 × 10^+01^(−)	2.1044 × 10^+01^(−)	2.1060 × 10^+01^(−)	2.1044 × 10^+0^1(−)	2.1060 × 10^+01^(−)	2.1012 × 10^+01^(−)	2.1062 × 10^+01^(−)	2.1003 × 10^+01^
9	5.5049 × 10^+01^(−)	3.7639 × 10^+01^(−)	2.4972 × 10^+01^(−)	1.2712 × 10^+01^(+)	2.0720 × 10^+01^(+)	5.9709 × 10^+01^(−)	2.7689 × 10^+01^(−)	2.6244 × 10^+01^
10	1.1534 × 10^+00^(−)	1.7926 × 10^+00^(−)	5.9149 × 10^−02^(−)	1.0602 × 10^−01^(−)	1.7241 × 10^−02^(+)	9.4477 × 10^−01^(−)	1.4780 × 10^−02^(+)	6.6495 × 10^−02^
11	5.6843 × 10^−14^(+)	5.6843 × 10^−14^(+)	2.0090 × 10^+01^(+)	1.4924 × 10^+01^(+)	5.2875 × 10^+01^(−)	1.0379 × 10^+00^(+)	7.4948 × 10^+01^(−)	2.2921 × 10^+01^
12	2.5041 × 10^+02^(−)	1.5262 × 10^+02^(−)	6.4672 × 10^+01^(−)	3.0614 × 10^+02^(−)	7.1792 × 10^+01^(−)	2.5256 × 10^+02^(−)	2.2941 × 10^+02^(−)	6.6662 × 10^+01^
13	3.1407 × 10^+02^(−)	2.5190 × 10^+02^(−)	1.3242 × 10^+02^(−)	3.1176 × 10^+02^(−)	1.2214 × 10^+02^(+)	2.4339 × 10^+02^(−)	2.8762 × 10^+02^(−)	1.2265 × 10^+02^
14	6.0810 × 10^+00^(+)	6.3847 × 10^+00^(+)	3.7529 × 10^+02^(+)	6.9829 × 10^+02^(−)	1.2919 × 10^+03^(−)	7.9588 × 10^+01^(+)	3.6695 × 10^+03^(−)	6.0000 × 10^+02^
15	1.1449 × 10^+04^(−)	6.7771 × 10^+03^(−)	5.0860 × 10^+03^(−)	3.6314 × 10^+03^(+)	8.5293 × 10^+03^(−)	1.0372 × 10^+04^(−)	1.1384 × 10^+04^(−)	5.1193 × 10^+03^
16	2.9382 × 10^+00(−)^	2.5038 × 10^+00^(−)	2.7308 × 10^−01^(−)	2.8265 × 10^+00^(−)	2.5942 × 10^+00^(−)	2.3831 × 10^+00^(−)	2.1997 × 10^+00^(−)	7.0698 × 10^−01^
17	5.0786 × 10^+01^(+)	5.0800 × 10^+01^(+)	7.2026 × 10^+01^(+)	3.1820 × 10^+02^(−)	1.1320 × 10^+02^(−)	5.9894 × 10^+01^(+)	1.3404 × 10^+02^(−)	6.8152 × 10^+01^
18	4.0051 × 10^+02^(−)	3.6304 × 10^+02^(−)	9.8335 × 10^+01^(−)	3.7049 × 10^+02^(−)	2.6744 × 10^+02^(−)	3.5631 × 10^+02^(−)	3.5551 × 10^+02^(−)	1.0152 × 10^+02^
19	6.6847 × 10^+00^(−)	8.9514 × 10^+00^(−)	4.0347 × 10^+00^(+)	4.5031 × 10^+00^(−)	5.0660 × 10^+00^(−)	9.5681 × 10^+00^(−)	1.0231 × 10^+01^(−)	3.4843 × 10^+00^
20	2.1634 × 10^+01^(−)	2.1809 × 10^+01^(−)	1.8929 × 10^+01^(−)	2.1551 × 10^+01^(−)	2.0609 × 10^+01^(−)	2.1465 × 10^+01^(−)	2.0438 × 10^+01^(−)	1.7868 × 10^+01^
21	2.0000 × 10^+02^(=)	2.0000 × 10^+02^(=)	2.0000 × 10^+02^(−)	2.0000 × 10^+02^(=)	2.0000 × 10^+02^(=)	2.0000 × 10^+02^(=)	2.0000 × 10^+02^(=)	2.0000 × 10^+02^
22	2.6406 × 10^+01^(+)	3.0189 × 10^+01^(+)	6.2665 × 10^+02^(+)	7.3845 × 10^+02^(−)	1.7683 × 10^+03^(−)	1.0261 × 10^+02^(+)	3.3192 × 10^+03^(−)	4.8452 × 10^+02^
23	1.2346 × 10^+04^(−)	9.3380 × 10^+03^(−)	4.8609 × 10^+03^(−)	3.1419 × 10^+03^(+)	8.5494 × 10^+03^(−)	1.1668 × 10^+04^(−)	1.1481 × 10^+04^(−)	4.9707 × 10^+03^
24	3.1693 × 10^+02^(−)	3.2104 × 10^+02^(−)	2.4601 × 10^+02^(−)	2.3006 × 10^+02^(+)	2.5158 × 10^+02^(−)	2.2959 × 10^+02^(+)	2.3078 × 10^+02^(+)	2.4894 × 10^+02^
25	3.5881 × 10^+02^(−)	3.6268 × 10^+02^(−)	3.0172 × 10^+02^(−)	2.8333 × 10^+02^(+)	2.8807 × 10^+02^(+)	3.3466 × 10^+02^(−)	2.8322 × 10^+02^(+)	2.9743 × 10^+02^
26	2.0453 × 10^+02^(−)	2.0252 × 10^+02^(−)	2.0021 × 10^+02^(−)	2.0010 × 10^+02^(+)	2.0008 × 10^+02^(+)	2.0071 × 10^+02^(−)	2.0004 × 10^+02^(+)	2.0019 × 10^+02^
27	1.5258 × 10^+03^(−)	1.6157 × 10^+03^(−)	7.9749 × 10^+02^(+)	6.9280 × 10^+02^(+)	8.2461 × 10^+02^(+)	1.5090 × 10^+03^(−)	7.3735 × 10^+02^(+)	9.2220 × 10^+02^
28	4.0000 × 10^+02^(=)	4.0000 × 10^+02^(=)	4.0000 × 10^+02^(=)	4.0000 × 10^+02^(=)	4.0000 × 10^+02^(=)	4.0000 × 10^+02^(=)	4.0000 × 10^+02^(=)	4.0000 × 10^+02^
−/=/+	20/4/4	20/4/4	20/2/6	12/5/11	14/4/10	19/2/7	14/5/9	−/−/−

**Table 4:** Comparison results of mean and standard deviation of 20-run fitness error among contrasted algorithms under CEC2013 test suite.

**50D**	**DE/best/1/bin**	**ODE/best/1/bin**	**CLPSO**	**SLPSO**
1	2.2737 × 10^−13^/0.0000 × 10^+00^(=)	2.2737 × 10^−13^/0.0000 × 10^+00^(=)	2.2737 × 10^−13^/0.0000 × 10^+00^(=)	2.2737 × 10^−13^/0.0000 × 10^+00^(=)
2	6.7630 × 10^+07^/1.4092 × 10^+07^(−)	8.1854 × 10^+07^/1.7030 × 10^+07^(−)	3.9702 × 10^+07^/7.0886 × 10^+06^(−)	8.9731 × 10^+05^/3.1582 × 10^+05^(=)
3	3.2967 × 10^+09^/1.8024 × 10^+09^(−)	4.4957 × 10^+09^/1.4925 × 10^+09^(−)	1.8074 × 10^+09^/9.5165 × 10^+08^(−)	1.1610 × 10^+07^/1.4090 × 10^+07^(+)
4	4.9175 × 10^+04^/4.9989 × 10^+03^(−)	5.7660 × 10^+04^/8.7742 × 10^+03^(−)	3.3408 × 10^+04^/6.0160 × 10^+03^(−)	3.3850 × 10^+04^/1.0284 × 10^+04^(−)
5	2.2737 × 10^−13^/3.6885 × 10^−14^(=)	1.9895 × 10^−13^/5.0507 × 10^−14^(=)	2.8990 × 10^−13^/5.8028 × 10^−14^(−)	1.9895 × 10^−13^/5.0507 × 10^−14^(=)
6	4.4426 × 10^+01^/7.7214 × 10^−01^(−)	4.5560 × 10^+01^/1.4691 × 10^+00^(−)	4.6402 × 10^+01^/7.0628 × 10^−01^(−)	4.3447 × 10^+01^/1.2356 × 10^−11^(+)
7	8.3113 × 10^+01^/1.0175 × 10^+01^(−)	8.8732 × 10^+01^/1.2892 × 10^+01^(−)	1.0165 × 10^+02^/8.5250 × 10^+00^(−)	5.9876 × 10^+00^/4.8864 × 10^+00^(+)
8	2.1127 × 10^+01^/3.5876 × 10^−02^(=)	2.1143 × 10^+01^/3.7340 × 10^−02^(=)	2.1143 × 10^+01^/3.7719 × 10^−02^(=)	2.1119 × 10^+01^/3.3008 × 10^−02^(=)
9	5.8061 × 10^+01^/1.8481 × 10^+00^(−)	5.0049 × 10^+01^/5.9272 × 10^+00^(−)	5.3471 × 10^+01^/2.5860 × 10^+00^(−)	1.8053 × 10^+01^/3.5882 × 10^+00^(+)
10	3.9408 × 10^+00^/2.0661 × 10^+00^(−)	6.3503 × 10^+00^/4.4398 × 10^+00^(−)	6.0611 × 10^+00^/1.4295 × 10^+00^(−)	2.6597 × 10^−01^/1.1229 × 10^−01^(−)
11	1.9402 × 10^+00^/1.6920 × 10^+00^(+)	1.9402 × 10^+00^/1.5970 × 10^+00^(+)	8.8107 × 10^−14^/2.9014 × 10^−14^(+)	3.4565 × 10^+01^/1.1287 × 10^+01^(=)
12	3.1874 × 10^+02^/2.7910 × 10^+01^(−)	2.5954 × 10^+02^/3.3785 × 10^+01^(−)	2.7169 × 10^+02^/2.8911 × 10^+01^(−)	3.4056 × 10^+02^/1.4672 × 10^+01^(−)
13	3.4943 × 10^+02^/1.7881 × 10^+01^(−)	3.1646 × 10^+02^/3.3786 × 10^+01^(−)	3.5904 × 10^+02^/3.9979 × 10^+01^(−)	3.3874 × 10^+02^/1.0522 × 10^+01^(−)
14	9.2118 × 10^+01^/9.8833 × 10^+01^(+)	5.4027 × 10^+01^/6.5389 × 10^+01^(+)	4.3188 × 10^+01^/1.1086 × 10^+01^(+)	1.1953 × 10^+03^/3.3024 × 10^+02^(−)
15	1.2980 × 10^+04^/6.5858 × 10^+02^(−)	1.1202 × 10^+04^/1.7749 × 10^+03^(−)	9.2360 × 10^+03^/5.1031 × 10^+02^(−)	1.2144 × 10^+04^/2.9152 × 10^+03^(−)
16	3.3028 × 10^+00^/2.1856 × 10^−01^(−)	3.2672 × 10^+00^/3.4818 × 10^−01^(−)	2.6884 × 10^+00^/2.9832 × 10^−01^(−)	3.3398 × 10^+00^/2.5719 × 10^−01^(−)
17	5.0939 × 10^+01^/2.1260 × 10^−01^(+)	5.1808 × 10^+01^/1.0106 × 10^+00^(+)	5.3451 × 10^+01^/5.9901 × 10^−01^(+)	3.5993 × 10^+02^/2.4006 × 10^+01^(−)
18	4.2249 × 10^+02^/1.4402 × 10^+01^(−)	3.9685 × 10^+02^/1.5608 × 10^+01^(−)	4.0577 × 10^+02^/2.3800 × 10^+01^(−)	3.9239 × 10^+02^/1.2010 × 10^+01^(−)
19	8.7896 × 10^+00^/7.5759 × 10^−01^(−)	1.0217 × 10^+01^/4.9098 × 10^−01^(−)	3.0401 × 10^+00^/4.7453 × 10^−01^(+)	6.3564 × 10^+00^/9.8914 × 10^−01^(−)
20	2.2341 × 10^+01^/2.9931 × 10^−01^(−)	2.2343 × 10^+01^/2.7942 × 10^−01^(−)	2.3215 × 10^+01^/5.3751 × 10^−01^(−)	2.2119 × 10^+01^/3.1389 × 10^−01^(−)
21	6.3252 × 10^+02^/4.5144 × 10^+02^(=)	7.4552 × 10^+02^/3.8638 × 10^+02^(=)	3.5629 × 10^+02^/1.7059 × 10^+02^(+)	8.3775 × 10^+02^/3.5207 × 10^+02^(=)
22	2.1962 × 10^+02^/5.4330 × 10^+02^(+)	8.2888 × 10^+02^/8.9069 × 10^+02^(=)	1.1107 × 10^+02^/8.2297 × 10^+01^(+)	1.3757 × 10^+03^/3.8553 × 10^+02^(−)
23	1.3292 × 10^+04^/4.4167 × 10^+02^(−)	1.1793 × 10^+04^/1.2168 × 10^+03^(−)	1.0989 × 10^+04^/7.4371 × 10^+02^(−)	1.2284 × 10^+04^/2.2400 × 10^+03^(−)
24	3.2829 × 10^+02^/8.3062 × 10^+00^(−)	3.3833 × 10^+02^/1.0558 × 10^+01^(−)	3.4471 × 10^+02^/8.4855 × 10^+00^(−)	2.5367 × 10^+02^/1.0552 × 10^+01^(+)
25	3.7028 × 10^+02^/7.0668 × 10^+00^(−)	3.7432 × 10^+02^/4.9628 × 10^+00^(−)	3.8750 × 10^+02^/7.9298 × 10^+00^(−)	2.9793 × 10^+02^/7.6039 × 10^+00^(+)
26	2.0754 × 10^+02^/1.6936 × 10^+00^(+)	2.1829 × 10^+02^/5.4942 × 10^+01^(+)	2.0422 × 10^+02^/1.0483 × 10^+00^(+)	3.2412 × 10^+02^/4.4937 × 10^+01^(+)
27	1.7343 × 10^+03^/8.7052 × 10^+01^(−)	1.7591 × 10^+03^/7.7658 × 10^+01^(−)	1.5672 × 10^+03^/4.9764 × 10^+02^(−)	7.9272 × 10^+02^/6.7907 × 10^+01^(+)
28	8.7540 × 10^+02^/1.1611 × 10^+03^(=)	7.1055 × 10^+02^/9.5585 × 10^+02^(=)	4.0000 × 10^+02^/3.8809 × 10^−05^(=)	4.0000 × 10^+02^/1.8070 × 10^−13^(+)
−/=/+	18/5/5	18/6/4	18/3/7	13/6/9
**50D**	**QUATRE/best**	**QUATRE/rand**	**QUATRE/target-to-best**	**AMP-QUATRE**
1	2.1600 × 10^−13^/5.0842 × 10^−14^(=)	4.5475 × 10^−14^/9.3312 × 10^−14^(+)	2.2737 × 10^−13^/0.0000 × 10^+00^(=)	2.2737 × 10^−13^/0.0000 × 10^+00^
2	1.0164 × 10^+06^/3.7357 × 10^+05^(=)	1.5023 × 10^+07^/4.6299 × 10^+06^(−)	5.5836 × 10^+05^/1.8107 × 10^+05^(+)	1.0360 × 10^+06^/3.4365 × 10^+05^
3	2.3504 × 10^+07^/2.4206 × 10^+07^(+)	4.4566 × 10^+07^/3.8021 × 10^+07^(=)	3.6782 × 10^+06^/4.3941 × 10^+06^(+)	5.9671 × 10^+07^/7.3033 × 10^+07^
4	1.3953 × 10^+02^/1.1877 × 10^+02^(+)	2.7389 × 10^+04^/4.9350 × 10^+03^(−)	4.8391 × 10^+01^/3.2542 × 10^+01^(+)	6.8604 × 10^+03^/1.8305 × 10^+03^
5	1.5348 × 10^−13^/5.5634 × 10^−14^(+)	5.3547 × 10^−12^/2.1671 × 10^−12^(−)	1.9895 × 10^−13^/5.0507 × 10^−14^(=)	2.1600 × 10^−13^/5.0842 × 10^−14^
6	4.5714 × 10^+01^/1.0138 × 10^+01^(=)	4.3448 × 10^+01^/2.3788 × 10^−04^(−)	4.3447 × 10^+01^/1.5166 × 10^−13^(+)	4.3741 × 10^+01^/1.2778 × 10^+00^
7	6.7584 × 10^+01^/2.9108 × 10^+01^(=)	4.7842 × 10^+01^/8.1591 × 10^+00^(=)	3.2089 × 10^+01^/1.3516 × 10^+01^(+)	5.0112 × 10^+01^/1.1419 × 10^+01^
8	2.1186 × 10^+01^/4.1762 × 10^−02^(−)	2.1130 × 10^+01^/4.0969 × 10^−02^(=)	2.1132 × 10^+01^/3.3735 × 10^−02^(=)	2.1129 × 10^+01^/4.3053 × 10^−02^
9	3.7688 × 10^+01^/9.3369 × 10^+00^(=)	6.2639 × 10^+01^/1.3582 × 10^+00^(−)	5.1629 × 10^+01^/1.2135 × 10^+01^(−)	3.5635 × 10^+01^/5.3692 × 10^+00^
10	4.8407 × 10^−02^/2.7521 × 10^−02^(+)	1.0614 × 10^+00^/4.4795 × 10^−02^(−)	5.1479 × 10^−02^/2.2316 × 10^−02^(+)	1.6877 × 10^−01^/9.1756 × 10^−02^
11	8.2337 × 10^+01^/1.8602 × 10^+01^(−)	3.2253 × 10^+00^/1.5308 × 10^+00^(+)	8.7687 × 10^+01^/6.7867 × 10^+00^(−)	3.2670 × 10^+01^/7.8026 × 10^+00^
12	1.7239 × 10^+02^/5.2618 × 10^+01^(−)	2.8889 × 10^+02^/1.8586 × 10^+01^(−)	2.6828 × 10^+02^/2.2955 × 10^+01^(−)	9.6453 × 10^+01^/1.9272 × 10^+01^
13	2.4279 × 10^+02^/6.2197 × 10^+01^(−)	3.2962 × 10^+02^/2.8567 × 10^+01^(−)	3.2354 × 10^+02^/1.9088 × 10^+01^(−)	1.8994 × 10^+02^/3.9348 × 10^+01^
14	2.0942 × 10^+03^/4.5566 × 10^+02^(−)	1.0795 × 10^+02^/2.0880 × 10^+01^(+)	4.0460 × 10^+03^/2.6165 × 10^+02^(−)	9.4145 × 10^+02^/2.6942 × 10^+02^
15	1.0621 × 10^+04^/1.3099 × 10^+03^(−)	1.2616 × 10^+04^/7.7705 × 10^+02^(−)	1.2556 × 10^+04^/4.3565 × 10^+02^(−)	6.7499 × 10^+03^/9.2335 × 10^+02^
16	3.2825 × 10^+00^/3.6056 × 10^−01^(−)	3.2270 × 10^+00^/3.4962 × 10^−01^(−)	3.1910 × 10^+00^/3.5035 × 10^−01^(−)	2.1841 × 10^+00^/6.6439 × 10^−01^
17	1.4570 × 10^+02^/2.2191 × 10^+01^(−)	6.3096 × 10^+01^/2.0368 × 10^+00^(+)	1.4326 × 10^+02^/6.5122 × 10^+00^(−)	8.4913 × 10^+01^/1.1647 × 10^+01^
18	3.3174 × 10^+02^/4.0023 × 10^+01^(−)	3.9469 × 10^+02^/1.6971 × 10^+01^(−)	3.8234 × 10^+02^/1.6744 × 10^+01^(−)	1.3042 × 10^+02^/1.7501 × 10^+01^
19	8.9865 × 10^+00^/2.3915 × 10^+00^(−)	1.1791 × 10^+01^/1.0069 × 10^+00^(−)	1.1623 × 10^+01^/6.9306 × 10^−01^(−)	5.7073 × 10^+00^/1.2275 × 10^+00^
20	2.1561 × 10^+01^/6.1393 × 10^−01^(−)	2.2261 × 10^+01^/2.7190 × 10^−01^(−)	2.1631 × 10^+01^/4.1090 × 10^−01^(−)	1.9466 × 10^+01^/9.0364 × 10^−01^
21	7.5331 × 10^+02^/4.6351 × 10^+02^(=)	3.3833 × 10^+02^/3.3784 × 10^+02^(+)	6.9616 × 10^+02^/4.2920 × 10^+02^(=)	8.3451 × 10^+02^/3.9226 × 10^+02^
22	2.7567 × 10^+03^/5.0416 × 10^+02^(−)	1.5228 × 10^+02^/3.7961 × 10^+01^(+)	4.0302 × 10^+03^/3.6030 × 10^+02^(−)	1.0156 × 10^+03^/3.1553 × 10^+02^
23	1.0749 × 10^+04^/1.2205 × 10^+03^(−)	1.2862 × 10^+04^/5.9890 × 10^+02^(−)	1.2310 × 10^+04^/4.3309 × 10^+02^(−)	7.3067 × 10^+03^/1.1271 × 10^+03^
24	2.8054 × 10^+02^/1.6244 × 10^+01^(=)	2.5232 × 10^+02^/2.0385 × 10^+01^(+)	2.5944 × 10^+02^/1.5156 × 10^+01^(+)	2.7678 × 10^+02^/1.4085 × 10^+01^
25	3.1143 × 10^+02^/1.3222 × 10^+01^(=)	3.6970 × 10^+02^/1.5311 × 10^+01^(−)	3.0858 × 10^+02^/1.7011 × 10^+01^(=)	3.1547 × 10^+02^/1.2938 × 10^+01^
26	3.7335 × 10^+02^/4.4089 × 10^+01^(=)	2.7737 × 10^+02^/1.1776 × 10^+02^(=)	3.4616 × 10^+02^/6.6582 × 10^+01^(+)	3.7563 × 10^+02^/4.3086 × 10^+01^
27	1.1743 × 10^+03^/2.1424 × 10^+02^(=)	1.8024 × 10^+03^/1.0782 × 10^+02^(−)	9.5415 × 10^+02^/1.3437 × 10^+02^(+)	1.1754 × 10^+03^/1.3799 × 10^+02^
28	1.1486 × 10^+03^/1.3303 × 10^+03^(=)	4.0000 × 10^+02^/3.5987 × 10^−09^(+)	8.4294 × 10^+02^/1.0819 × 10^+03^(=)	1.1617 × 10^+03^/1.3536 × 10^+03^
−/=/+	13/11/4	16/4/8	13/6/9	−/−/−

**Table 5:** Parameter settings for simulation.

Simulation Parameters	Parameters Settings
Sensing region area	100 m × 100 m
Total number of sensor nodes	100–400
Communication range	15–40 m
Percentage of anchor nodes	5–40%
Initial population size	20
Maximum generations	100

**Table 6:** Comparison of location errors of the applied AMG-QUATRE with the other methods for different anchor nodes.

Anchor Nodes	5	10	15	20	25	30	35	40	Avg
DV-Hop	0.495	0.4227	0.423	0.349	0.3513	0.346	0.322	0.3213	0.378788
Hyperbolic-DV-Hop	0.4847	0.3716	0.3641	0.3382	0.3004	0.3185	0.3023	0.2834	0.3454
PSO-DV-Hop	0.4855	0.2979	0.2554	0.2289	0.2004	0.2001	0.1931	0.188	0.256163
DE-DV-Hop	0.4423	0.2634	0.2525	0.2207	0.1995	0.1997	0.1937	0.1849	0.244588
AMG-QUATRE-DV-Hop	0.4255	0.2605	0.2525	0.2209	0.1993	0.1995	0.1934	0.1855	0.242138

**Table 7:** Comparison of location errors of the applied AMG-QUATRE with the other methods for different communication ranges.

Communication Range	15	20	25	30	35	40	Avg.
DV-Hop	0.5286	0.349	0.3219	0.2968	0.3149	0.3002	0.3519
Hyperbolic-DV-Hop	0.5603	0.3382	0.3	0.2546	0.2931	0.2634	0.334933
PSO-DV-Hop	0.2919	0.2286	0.2081	0.203	0.2053	0.1945	0.2219
DE-DV-Hop	0.281	0.2204	0.2042	0.1909	0.2038	0.194	0.215717
AMG-QUATRE-DV-Hop	0.2806	0.2209	0.205	0.1911	0.2037	0.1939	0.215867

**Table 8:** Comparison of the applied AMG-QUATRE with the other methods by different sensor nodes.

Sensor Nodes	100	150	200	250	300	350	400	Avg.
DV-Hop	0.4645	0.3711	0.349	0.3771	0.3513	0.3022	0.2887	0.3577
Hyperbolic-DV-Hop	0.3745	0.3368	0.3382	0.3067	0.3166	0.2998	0.2818	0.322057
PSO-DV-Hop	0.3106	0.3178	0.2342	0.2303	0.1887	0.1784	0.1741	0.233443
DE-DV-Hop	0.2967	0.2777	0.2205	0.2234	0.1828	0.1769	0.1741	0.221729
AMG-QUATRE-DV-Hop	0.2979	0.284	0.2209	0.2225	0.1816	0.1773	0.1742	0.222629
